# A Single DBS-Lead to Stimulate the Thalamus and Subthalamus: Two-Story Targets for Tremor Disorders

**DOI:** 10.3389/fnhum.2022.790942

**Published:** 2022-01-24

**Authors:** Jumpei Sugiyama, Hiroki Toda

**Affiliations:** Department of Neurosurgery, Tazuke Kofukai Medical Research Institute and Kitano Hospital, Osaka, Japan

**Keywords:** tremor, deep brain stimulation, ventral intermediate nucleus, ventral posterior oralis nucleus, thalamus, subthalamus

## Introduction

The recent development of magnetic resonance-guided focused ultrasound highlights the clinical significance of the stereotactic surgery of the thalamus including deep brain stimulation (DBS). Essential tremor and parkinsonian tremor respond well to DBS of the ventral intermediate nucleus (Vim) of the thalamus (Cury et al., 2017; Dallapiazza et al., 2019). Other tremor disorders, such as dystonic tremor, multiple sclerosis tremor, and Holmes' tremor, are treated well with stimulation of the ventral oralis anterior (Voa) and posterior (Vop) nuclei of the thalamus (Katayama et al., 2005; Foote et al., 2006; Cury et al., 2017; Mongardi et al., 2020). The Voa/Vop is also chosen as a DBS target when an intraoperative Vim stimulation trial is not satisfactory (Katayama et al., 2005; Molnar et al., 2005). In such cases, the DBS electrode is placed slightly anteriorly to stimulate the Voa/Vop. The Vim and Voa/Vop are adjacent primary targets to treat various tremor disorders.

The subthalamus, located beneath the ventral tier of the thalamic motor nuclei, also contains targets to control tremor disorders. The known surgical targets within the subthalamus are the caudal part of the zona incerta (Zi, cZi) (Fytagoridis et al., 2012; Eisinger et al., 2018), prelemniscal radiation (Raprl) (Kitagawa et al., 2000; Castro et al., 2015), and Fields H of Forel (Contreras Lopez et al., 2016; Fleury et al., 2016). The cZI and Raprl are often referred as the posterior subthalamic area (PSA) (Blomstedt et al., 2009).

Some refractory tremors may need two DBS-leads per hemisphere to stimulate dual- targets within the thalamus and subthalamus (Foote et al., 2006; Kobayashi et al., 2014). While two leads can generate a complex volume of tissue activated (VAT) and stimulate both pallido-thalamic and cerebello-thalamic projections, the multiple-lead insertion may increase the risk of surgical morbidity and dual internal pulse generators may be implanted per side.

The technological advances in the last 10 years include new DBS-lead designs as eight-contact directional or long span lead and programming technologies as interleaved or independent current control systems. With these advances, we can design complex volumes of tissue activated and apply these technologies for a single DBS-lead dual-target approach. This opinion article describes the benefit of a single DBS-lead trajectory going through two-story targets made up of the ventral motor nuclei of the thalamus (upstairs) and subthalamus (downstairs).

## DBS Target and Technology

### DBS Target Anatomy

#### Ventral Motor Nuclei of the Thalamus

**Figure 1:**
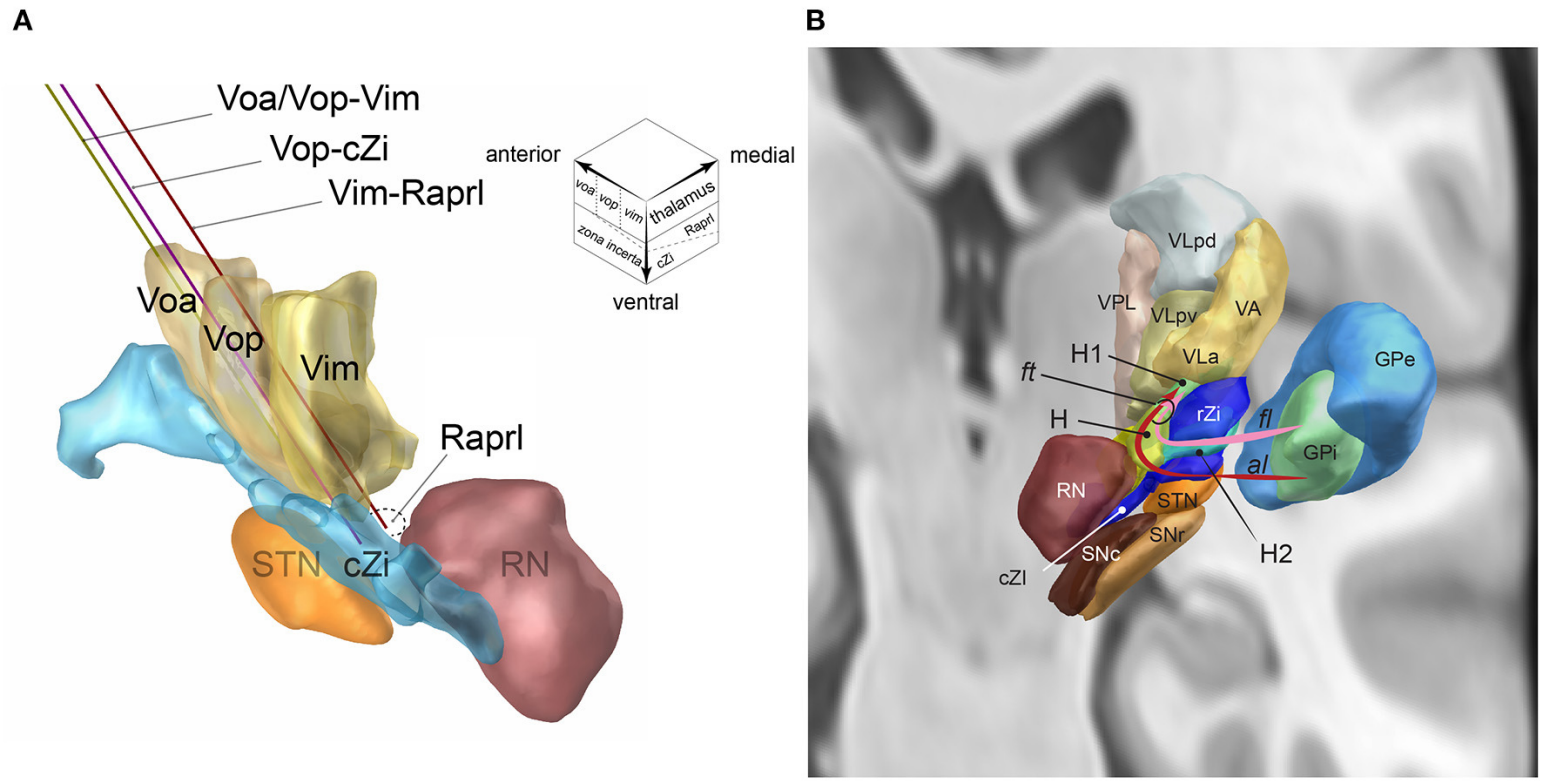
**(A)** The single DBS-lead dual-target trajectories going through two-story targets made of the ventral thalamic nuclei (upstairs) and posterior subthalamic area (downstairs, inset): Voa/Vop-Vim (yellow), Vop-cZi (blue), and Vop-Raprl (brown). Voa, nucleus ventralis oralis anterior; Vop, nucleus ventralis oralis posterior; Vim, nucleus ventralis intermedius; cZi, caudal zona incerta; Raprl, prelemniscal radiation; RN, nucleus ruber; STN, nucleus subthalamicus. The upper right voxel scheme illustrates the two-story structure of ventral motor nuclei of the thalamus (upstairs) and subthalamus (downstairs). **(B)** Fasciculus lenticularis (*fl*) leaves from dorsal globus pallidus interna (GPi), enters the field H2 of Forel (H2) between rostral zona incerta (rZi) and the subthalamic nucleus (STN), joined with ansa lenticularis(*al*) from ventral GPi in the field H of Forel (H), rostral to the red nucleus (RN), and form the fasciculus thalamicus (*ft*) in the field H1 of Forel (H) then projects to the VA, and VLa nuclei of the thalamus. GPe, GP externa; SNc substantia nigra pars compacta; SNr, SN pars reticulata; VLpd and VLpv, ventrolateral posterior dorsal and ventral nuclei of the thalamus; VPL, ventroposterolateral nucleus of the thalamus [Reproduced from Toda et al. (2021) with permission from the Igaku-Shoin, Ltd., Japan]. The figure components are generated using Lead-DBS (https://www.lead-dbs.org/).

Above the plane containing the anterior commissure (AC) and posterior commissure (PC), the ventral motor nuclei of the thalamus are arrayed as Voa, Vop, and Vim from the rostral to caudal direction (Figure 1A). The ventral tier of these thalamic ventral nuclei is around the AC–PC plane. The Vim is about 5–8 mm anterior to the PC (Guiot et al., 1961; Ohye et al., 1982; Jankovic et al., 1995) and is located anterior to the ventral caudal nucleus (Vc) which receives the sensory spinothalamic projection. The anteroposterior length of the Vim is 2–3 mm (Schaltenbrand et al., 1977). The Vim mainly receives the cerebellar projection via the fasciculus cerebello-thalamicus contained in the Raprl. A minor portion of the fasciculus thalamics from the pallidal component projects into the Vim (Gallay et al., 2008).

The Voa and Vop are located anterior to the Vim. The anteroposterior lengths of the Voa and Vop are both 1.5–2 mm (Schaltenbrand et al., 1977). The Voa/Vop receives most pallido-thalamic projection via the fasciculus thalamicus (*ft*, Figure 1B) (Gallay et al., 2008). The *ft* are made up of the ansa lenticularis (*al*) and fasciculus lenticularis (*fl*). The *al* originates from the ventral globus pallidus interna (GPi). The *al* is ventral and medial of the subthalamic nucleus (STN) and makes a turn (*ansa*) to go over the STN. The *fl* originates from the dorsal GPi. The *fl* goes between the dorsal STN and ventral Zi, the field H2 of Forel (Forel H2). The *al* and *fl* go through the prerubral field H of Forel (Forel H) and merge to become the *ft*. The *ft* goes through field H1 of Forel (Forel H1) and then enters the Voa/Vop (Gallay et al., 2008).

#### Subthalamus

The subthalamus is located underneath the AC-PC plane and beneath the ventral border of the thalamic motor nuclei (Parent and Carpenter, 1996). The subthalamus can be divided as the anterior and posterior subthalamic areas (Blomstedt et al., 2009). In the anterior subthalamic area, there are four layers from the dorsal to ventral direction: (i) Forel H1(including the *ft* as described above), (ii) rostal Zi, (iii) Forel H2 (including *fl*), and (iv) STN. The posterior subthalamic area (PSA) consists of the cZi and Raprl (Figure 1A) (Blomstedt et al., 2009). The PSA is bounded by the STN (anterior), medial lemniscus (posterior), red nucleus (medial), and internal capsule and the Vc of the thalamus (lateral). The Zi has broad bidirectional projections to the cerebellum, brainstem reticular formation, pedunculopontine nucleus, substantia nigra pars reticulata, GPi, superior colliculus, cerebral cortex, and spinal cord. Most Zi neurons are inhibitory GABAergic neurons and include excitatory glutamatergic and dopaminergic neurons (Wang et al., 2020). The Raprl is constituted of cerebellothalamic tract and mesencephalo-reticulo-thalamic tract (Gallay et al., 2008; Blomstedt et al., 2009, 2012; Ramirez-Zamora et al., 2016; Nowacki et al., 2018).

### DBS Trajectory

A single DBS-lead trajectory can align the thalamic and subthalamic targets by adjusting the entry point and trajectory angle. The combinations of the thalamic and subthalamic targets include Voa/Vop–Vim, Vop–subthalamus, and Vim–subthalamus (Figure 1A). The posterior subthalamic area can be localized according to the red nucleus. Slight adjustment of the mediolateral angle may choose the cZi in the lateral PSA and the Raprl in the posteromedial PSA (inset of Figure 1A).

## Discussion

The dual-target approach can be a useful strategy to treat refractory tremors such as Holmes' tremor (Foote and Okun, 2005; Foote et al., 2006; Kobayashi et al., 2014; Artusi et al., 2018; Parker et al., 2020). Holmes' tremor manifests a mixed presentation of basal ganglia tremor via the pallido-thalamic projection and cerebellar tremor via the cerebello-thalamic projection (Deuschl et al., 2001). Therefore, Holmes' tremor may be treated with dual-target strategy stimulating the Voa/Vop and the Vim nuclei by using dual DBS-lead insertion (Foote and Okun, 2005; Foote et al., 2006; Kobayashi et al., 2014; Artusi et al., 2018; Parker et al., 2020).

A single DBS-lead dual-target approach is an alternative to the dual DBS-lead insertion. Several groups, including the authors, utilize the single DBS-lead approach to stimulate both striato-pallido-thalamic and cerebello-thalamic projections for patients with essential tremor (Barbe et al., 2018; Bot et al., 2018; Dos Santos Ghilardi et al., 2018), Holmes' tremor (Toda et al., 2017b), and atypical tremor (Toda et al., 2017a; Nakajima et al., 2021). Placing the single DBS-lead across the Vop/Vim border is the simplest option to stimulate both the striato-pallidal and cerebellar pathways (Nakajima et al., 2021). The Vim (Toda et al., 2017a; Barbe et al., 2018; Bot et al., 2018; Dos Santos Ghilardi et al., 2018) and the Voa/Vop nuclei (Toda et al., 2017b; Parker et al., 2020) are also aligned with the PSA on a single trajectory (Figure 1A). As traditional and empirical surgical nuances for Vim electrode placement, some surgeons insert the electrode slightly deeper in the ventral border of the thalamus to stimulate subthalamus as well as Vim. Nowadays, the long DBS octrode can traverse the ventral tier of the thalamus and subthalamus (Dos Santos Ghilardi et al., 2018). A recent trial by the University of Cologne (Barbe et al., 2018) compares stimulation of the Vim and PSA by inserting the single lead traversing both targets and concludes the equal and possibly more efficient effect of PSA-DBS than Vim-DBS. The best electrode to control tremors may vary among patients. Therefore, the clinician can compare the effects of the Vim stimulation and the PSA stimulation by selecting the electrode along the single DBS lead (Barbe et al., 2018; Dos Santos Ghilardi et al., 2018).

In addition, the interleaved (Toda et al., 2017a,b) or multiple independent current control system can expand the benefit of single lead stimulation by stimulating multiple electrodes along the lead. Therefore, even with the single lead insertion, patient and clinician can have options for stimulating multiple targets to control refractory tremors with no additional DBS lead.

Furthermore, PSA components can be stimulated selectively when the lead is placed at the border of the Raprl and cZi. The directional lead can also stimulate the cZi, Raprl, or both by choosing directed electrodes. Further complex insertion is possible by careful preoperative imaging examination. The recent postoperative imaging technology as Lead-DBS [https://www.lead-dbs.org/, (Horn et al., 2019)] can be a useful investigational tool to estimate and predict the deep brain structure along the DBS lead.

The single DBS-lead dual-target approach can be applied bilaterally to control tremors on both limbs, especially for patients with essential tremor (Barbe et al., 2018; Bot et al., 2018; Dos Santos Ghilardi et al., 2018). In addition, the bilateral dual-target strategy can be useful for midline tremor. The authors utilized bilateral Vim-PSA DBS leads to control tongue tremor and atypical parkinsonism which manifests as rigidity of the bilateral limbs (Toda et al., 2017a).

Application of the single-lead dual target approach is limited by the presence of cortical vein, lateral ventricle, and intraparenchymal vessels along the trajectory. Therefore, the detailed planning of the trajectory and target coordinates is necessary for single lead insertion for dual target.

In conclusion, the single DBS-lead dual-target stimulation within the ventral tier of the motor thalamic nuclei and the subthalamus can be a useful solution for refractory tremor.

## Author Contributions

All authors contributed to manuscript conception, draft, writing, revision, read, and approved the submitted version.

## Funding

HT received Grant-in-Aid for Scientific Research (21K09116) for this research from Japan Society for the Promotion of Science.

## Conflict of Interest

The authors declare that the research was conducted in the absence of any commercial or financial relationships that could be construed as a potential conflict of interest.

## Publisher's Note

All claims expressed in this article are solely those of the authors and do not necessarily represent those of their affiliated organizations, or those of the publisher, the editors and the reviewers. Any product that may be evaluated in this article, or claim that may be made by its manufacturer, is not guaranteed or endorsed by the publisher.
